# Endoscopic submucosal dissection for large colorectal epithelial neoplasms: A single center experience in north China

**DOI:** 10.1097/MD.0000000000007967

**Published:** 2017-09-08

**Authors:** Xin Xu, Tao Wang, Zhongqing Zheng, Xin Chen, Wentian Liu, Chao Sun, Bangmao Wang

**Affiliations:** a Department of Gastroenterology and Hepatology; b Tianjin Institute of Digestive Disease, Tianjin Medical University General Hospital, Heping District, Tianjin, China.

**Keywords:** colorectal neoplasms, endoscopic submucosal dissection, risk factors, submucosal fibrosis

## Abstract

Colorectal endoscopic submucosal dissection (ESD) is a technically difficult procedure with a higher risk of complications, especially for large colorectal epithelial neoplasms. This study aimed to report our experience and clinical outcomes, and to estimate the factors associated with incomplete resection and complications.

One hundred forty one colorectal epithelial neoplasms in 130 consecutive patients treated by ESD at the endoscopy center of Tianjin Medical University General Hospital from January 2013 to January 2016 were included. Factors associated with the incomplete resection and perforation were evaluated.

The mean colorectal epithelial neoplasm size was 26.5 ± 9.5 (15.0–60.0) mm, the mean procedure time of colorectal ESD was 76.1 ± 48.7 (36.5–195.0) minutes. The en bloc resection rate, the en bloc R0 resection rate, and the curative resection rate, were 93.6% (132/141), 91.5% (129/141), and 88.7% (125/141), respectively. Perforation during colorectal ESD occurred in 7 patients (4.9%), postoperative bleeding occurred in 4 patients (2.8%). There was no recurrence occurred in all patients during follow periods of 13.2 ± 8.6 (6.0–36.0) month. Submucosal fibrosis was the only independent factor related to both incomplete resection (odds ratio [OR] 12.425; 95% confidence interval [CI] 2.501–61.734; *P* = .002) and perforation (OR 10.646; 95%CI 1.188–95.421; *P* = .035) of colorectal ESD.

Colorectal ESD is a safe and effective technique for en bloc resection of large colorectal epithelial neoplasms. Submucosal fibrosis was independently related to incomplete resection and perforation.

## 1 Introduction

Endoscopic submucosal dissection (ESD), which was originally invented for early gastric cancer, has facilitated the en bloc resection with tumor-free margins of large superficial tumors in recent years.^[1]^ Due to the widespread acceptance of gastric ESD, along with the continuous improvement of technique devices for ESD, the colorectal ESD has become increasingly common and was exactly useful in en bloc resection for large colorectal superficial tumors.^[2,3]^ Suitable lesions for endoscopic treatment include not only early colorectal carcinomas but also many types of precarcinomatous adenomas. Recently, the Japan Gastroenterological Endoscopy Society has established a guideline^[4]^ for colorectal ESD and endoscopic mucosal resection (EMR) in 2015, which standardized the diagnostic and therapeutic strategies and stipulations of endoscopic procedures for colorectal tumors.

Colorectal ESD is a technically difficult procedure because the colonic wall is thin and endoscopic maneuverability is limited caused by colonic flexure and extensibility. Colorectal ESD has been reported to achieve a higher en bloc resection rate and lower recurrence rate than EMR, meanwhile, enable detailed pathological evaluation with less invasive treatment.^[5,6]^ However, colorectal ESD resulted in an increased risk of perforation because of the complex and challenging nature of the technique. Numerous previous studies investigated the risk factors for perforation of colorectal ESD.^[7–9]^ Submucosal fibrosis in particular is not only 1 major contributor to perforation during colorectal ESD, but also 1 major cause for increasing the probability of incomplete resections.^[10,11]^

As colorectal ESD has been widely available to treat the increasing number of epithelial neoplasms in Eastern nations, therefore, we aimed to report our experience, clinical outcomes, and prognosis of large colorectal epithelial neoplasms treated by ESD in a setting of north China. Furthermore, we aimed to estimate the potential factors of incomplete resection and complications for colorectal ESD procedures.

## 2 Patients and methods

### 2.1 Patients

We retrospectively reviewed the electronic database and medical charts of 141 colorectal epithelial neoplasms treated by ESD in 130 consecutive patients from January 2013 to January 2016. This study was carried out at the endoscopy center of Tianjin Medical University General Hospital, Tianjin, China, and was approved by the institutional ethical committee.

### 2.2 Indications for colorectal ESD

An initial endoscopy, chromoendoscopy, endoscopic ultrasonography (EUS) (model SP701; Fijinon, Omiya, Japan), and abdominal contrast-enhancement computed tomography (CT) were used concurrently for all patients to predict the invasion depth and metastasis before colorectal ESD procedures.

Indications for colorectal ESD are as follows: depth of invasion limited to the mucosa or submucosa with a noninvasive pattern on chromoendoscopy and EUS. Large lesions in which en bloc resection using EMR are difficult. Lesions with submucosal fibrosis caused by previous endoscopic treatment or biopsy. Local residual of early carcinoma after endoscopic resection.

Exclusion criteria included lesions with evidence of submucosal deep invasion diagnosed by chromoendoscopy and EUS; lesions with evidence of local or distant metastasis diagnosed by contrast-enhancement CT; lesions of submucosal tumor; lesions with inflammatory bowel disease.

### 2.3 ESD procedure

All patients were admitted to hospital and underwent fasting and bowel preparation of drinking 2 L of polyethylene glycol solution the day before the procedure. The patients were generally anesthetized by intravenous injection of midazolam and/or propofol. The cardiopulmonary function was monitored by an anesthetist following endotracheal intubation in an endoscopy unit. Intravenous scopolamine was administered to reduce colonic movements. All ESD procedures were performed by 3 experienced endoscopists (ZZ, TW, XC).

Colorectal ESD was performed with carbon dioxide insufflation, using a single-channel endoscope (model GIF-Q260J; Olympus, Tokyo, Japan). A transparent hood (model MH-593; Olympus) was attached to the tip of the endoscope for a good visualization. The electrosurgical current was applied using an electrosurgical generator (model ICC200; Erbe, Tubingen, Germany). The tumor was marked with several dots around the lesion using argon plasma coagulation (APC) (model APC300; Erbe). A saline solution combined with epinephrine, glycerin fructose, and methylene blue was injected into the submucosa with an injection needle (model NET 2522-G4; Endo-Flex GmbH, Voerde, Germany). A Dual-knife (KD 650U; Olympus) was used for circumferential incision along the margin of the targeted lesion. A Dual-knife, or a hybrid knife (JET2; Erbe), or an insulated-tip knife (ITknife2 model KD-611L; Olympus), was used to dissect the surrounding tissue at the level of the deepest submucosal layer and shell the connective muscular fibers and stalks along the capsule of the tumor. An electrosurgical knife, or a snare (model NOE 342217-G; Endo-Flex GmbH) sometimes was used to remove the tumor at the root completely. When perforation occurred during the procedure, metallic clips (model HX-610–135L; Olympus) were carried out as soon as possible, regardless of the location. Coagulation (APC) or clipping was carried out for bleeding associated with the procedure.

### 2.4 Histopathological assessment

The specimen was pinned on a cork sheet flatly then fixed with 10% formaldehyde solution. The macroscopic tumor type of colon superficial neoplastic lesions was classified according to the Paris endoscopic classification.^[12]^ Lesions were classified as protruding large tumors and laterally spreading tumors (LSTs). LSTs are classified into granular type (LST-G) and nongranular type (LST-NG). Tumor locations were grouped into cecum, ascending colon, transverse colon, descending colon, sigmoid colon, and rectum. Histopathological assessment was evaluated in accordance with the Vienna classification, grouped into adenoma, intramucosal carcinoma, SM1 (<1000 mm) carcinoma, and SM2 (>1000 mm) carcinoma.^[2–4]^

### 2.5 Definitions of evaluation index

Submucosal fibrosis was classified into 3 groups on the basis of findings obtained at the time of submucosal dissection after the injection. Criteria were as follows^[13]^: no fibrosis (F0), manifested as a blue transparent layer; mild fibrosis (F1), appearing as a white web-like structure in the blue submucosal layer; and severe fibrosis (F2), manifested as a white muscle-like structure without a blue transparent layer in the submucosal layer.

En bloc resection was defined as a 1-piece resection of an entire lesion as observed endoscopically. En-bloc R0 resection was defined as tumor removal in a 1-piece resection with tumor-free lateral and vertical margins. Curative resection was obtained when tumor removal in a 1-piece resection with tumor-free lateral and vertical margins, and there was no submucosal invasion of 1000 mm or more, lymphatic invasion, vascular involvement, or poorly differentiated component.

Surgery was recommended when the tumor was diagnosed as invasive carcinoma with deep submucosal invasion (>1000 mm) or exhibited risk factors for lymph node metastasis, vascular involvement, budding, or poor differentiation.^[4,10,14,15]^ Additional endoscopic treatment was recommended for the patient who could not suffer the risk of surgery, considering the body age, life expectancy, and comorbidities.

Perforation during an ESD was defined as immediate perforation which was deemed as a full-thickness defect during the ESD. Delayed perforation was defined as any perforation occurring after completion of the procedure.^[4,7]^ Postoperative bleeding was defined as clinical evidence of bleeding manifested by hematochezia within 14 days after the procedure that required endoscopic hemostasis.^[4,16]^

### 2.6 Follow up

All patients received follow-up endoscopy at 3, 6, and 12 months after the initial ESD therapy during the first year and thereafter annually. Any suspicious lesions were confirmed by biopsy.

### 2.7 Statistical analysis

Continuous variables were tested by using independent 2-side *t* test and categorical variables were analyzed by using the chi-squared test or Fisher exact test as appropriate. Risk factors were analyzed by univariate logistic regression analysis. Factors with a *P* value <.1 were included into the multiple logistic regression analysis. SPSS software version 22.0 (SPSS Inc., Chicago, IL) was used for statistical analysis. A *P* value <.05 was considered to indicate statistical significance.

## 3 Results

### 3.1 Clinicopathological characteristics of colorectal ESD

The clinicopathological characteristics of colorectal ESD were shown in Table 1. The mean age of the patients was 63.79 ± 11.26 (range, 30–86) years. The male to female ratio was 71:59. The mean neoplasm size was 26.5 ± 9.5 (range, 15.0–60.0) mm. Lesions were located in the rectum (24.1%), sigmoid colon (18.4%), descending colon (26.2%), transverse colon (7.8%), ascending colon (19.1%), and cecum (4.2%), respectively. Macroscopic types demonstrated, 30 LST-G (21.3%), 48 LST-NG (34.0%), and 63 protruded (44.7%), respectively. Histologically, there were 47 low-grade adenomas (33.3%), 54 high-grade adenomas (38.3%), 24 intramucosal adenocarcinomas (17.0%), 11 adenocarcinomas with superficial submucosal invasion (<1000 μm, 7.8%), and 5 adenocarcinomas with deep submucosal invasion (>1000 μm, 3.5%). The mean procedure time of colorectal ESD was 76.1 ± 48.7 (range, 36.5–195.0) minutes. The mean follow period after colorectal ESD was 13.2 ± 8.6 (range 6.0–36.0) month.

**Table 1:**
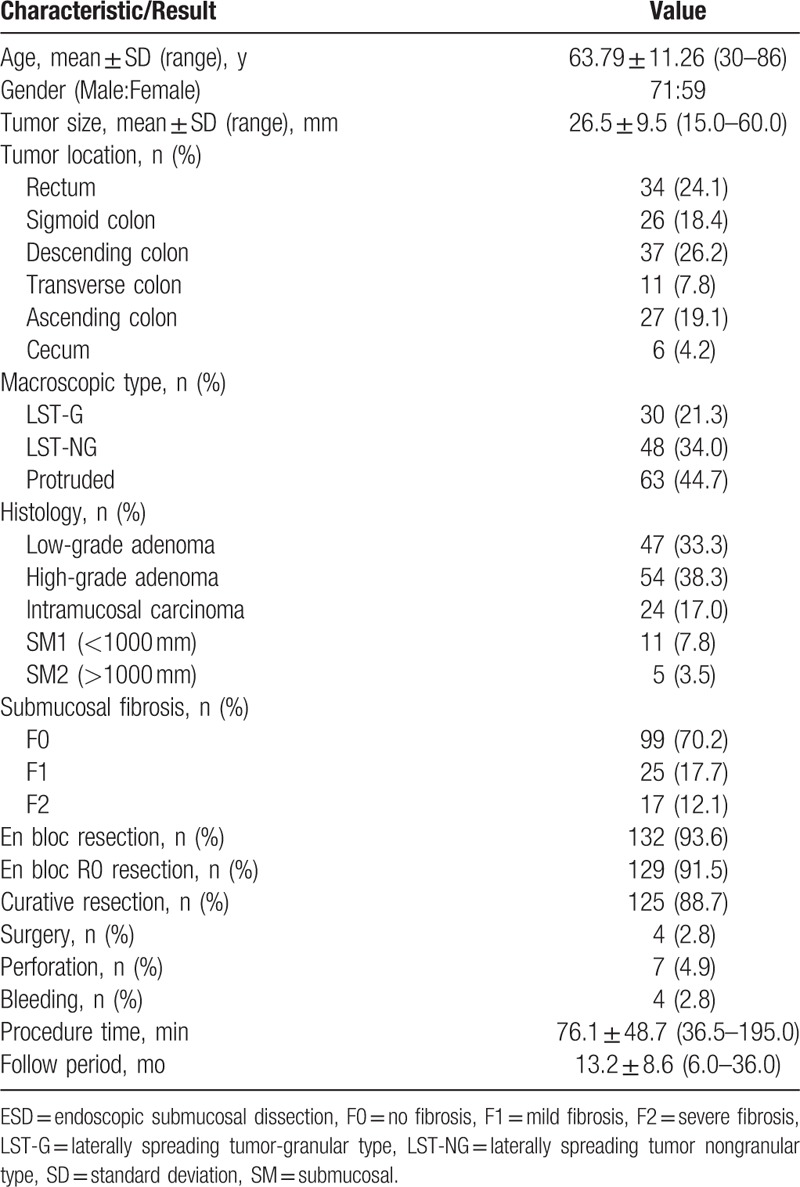
Clinicopathologic characteristics and treatment results of colorectal ESD.

### 3.2 Treatment results of colorectal ESD

The treatment results of colorectal ESD procedures were shown in Table 1. As a whole, the en bloc resection rate, the en bloc R0 resection rate, and the curative resection rate were 93.6% (132/141), 91.5% (129/141), and 88.7% (125/141), respectively. Of the 40 adenocarcinomas, 24 (60%) were intramucosal adenocarcinomas and 16 (40%) were submucosal adenocarcinomas. The additional surgery rate after colorectal ESD was 2.8% (4/141), and there was no recurrence occurred in all patients during follow periods. As shown in Table 2, 9 cases of adenocarcinoma were regarded as noncurative resection because of piecemeal resection or positive resection margins, together with deeper submucosal invasion (>1000 μm), lymphatic invasion, or vascular involvement. Additional surgical resection was recommended, and 4 of these patients have undergone additional surgical resection with lymphadenectomy immediately after colorectal ESD. Another patient has undergone additional ESD for piecemeal resection and positive vertical resection margins. While the remaining 4 patients refused surgical intervention were under intensively followed-up, and there was no recurrence occurred during their follow periods.

**Table 2:**
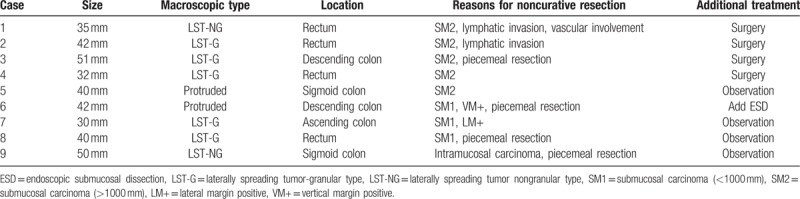
Characteristics of adenocarcinoma cases with noncurative resection.

Submucosal fibrosis was identified in 42 (29.8%) lesions during colorectal ESD, including 25 (17.7%) mild fibrosis (F1) lesions and 17 (12.1%) severe fibrosis (F2) lesions. Univariate logistic regression analysis revealed that tumor size exceeding 30 mm (odds ratio [OR] 3.864; 95% confidence interval [CI] 1.102–13.542; *P* = .035), and submucosal fibrosis (OR 15.156; 95% CI 3.154–72.842; *P* = .001) were significantly associated with the incomplete resection of colorectal ESD (Table 3). Multivariate logistic regression analysis identified that submucosal fibrosis (OR 12.425; 95% CI 2.501–61.734; *P* = .002) was the only independent factor related to incomplete resection of colorectal ESD.

**Table 3:**
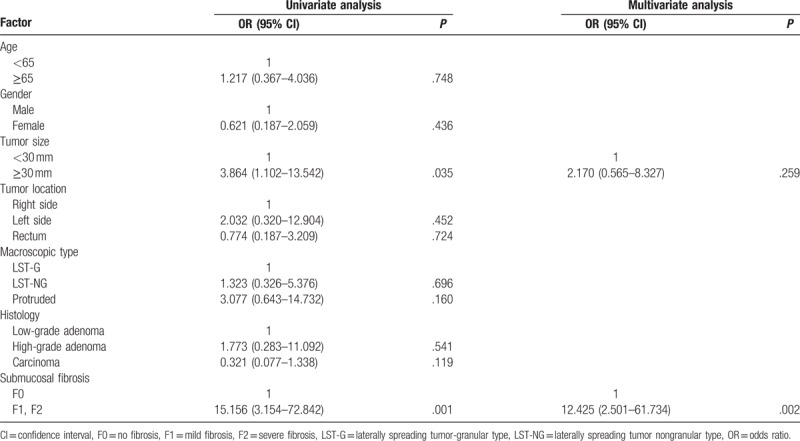
Univariate and multivariate analysis for factors affecting the incomplete resection.

### 3.3 Complications of colorectal ESD

Perforation occurred in 7 patients (4.9%). All of them were detected during the ESD procedures and were successfully closed by endoscopic clipping. After closure of the perforation, all patients were hospitalized to receive fasting and intravenous antibiotic conservative treatment, avoiding of emergency surgery. There was no case of delayed perforation in all colorectal ESD procedures. Postoperative bleeding occurred in 4 patients (2.8%), and all were cured conservatively by coagulation or clipping, without blood transfusion or surgical intervention. Univariate logistic regression analysis revealed that tumor size exceeding 30 mm (OR 11.478; 95% CI 1.341–98.227; *P* = .026), and submucosal fibrosis (OR 16.333; 95% CI 1.900–140.386; *P* = .011) were significantly associated with the perforation of colorectal ESD (Table 4). Multivariate logistic regression analysis identified that submucosal fibrosis (OR 10.646; 95% CI 1.188–95.421; *P* = .035) was the only independent factor related to perforation of colorectal ESD.

**Table 4:**
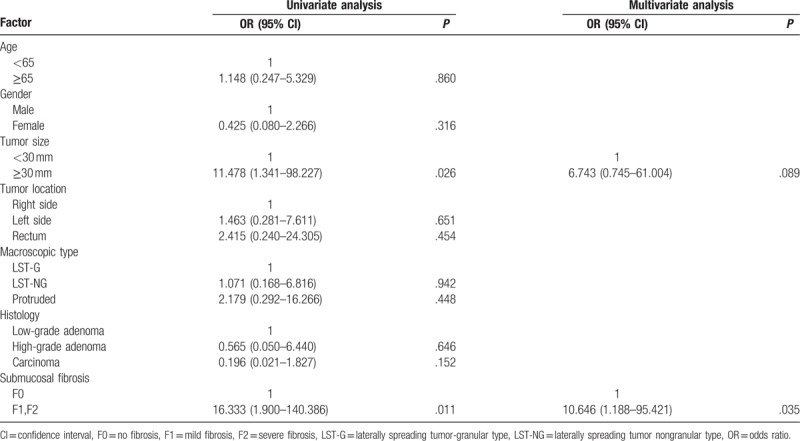
Univariate and multivariate analysis for factors affecting the perforation.

## 4 Discussion

Colorectal ESD has been widely available to treat the increasing number of epithelial neoplasms worldwide, especially in the Eastern nations. However, there are few clinical studies on ESD outcomes for large colorectal neoplasms in China. In this single-center retrospective study presented, colorectal ESD outcomes for large colorectal neoplasms have been reported. We confirmed that the colorectal ESD was a safe and efficient procedure regarding en bloc resection of large superficial colorectal neoplasms. We also demonstrated that the submucosal fibrosis was an independent factor related to incomplete resection and perforation.

Generally, the en bloc resection rate, the en bloc R0 resection rate, the curative resection rate were 93.6% (132/141), 91.5% (129/141), and 88.7% (125/141), respectively, in the presented study. Previous multicenter study from Asian countries revealed that ESDs were amenable to colorectal tumors with an overall en bloc resection rate and curative resection rate of 95.4% and 89.1%, respectively.^[3]^ Another latest systematic review summarized the mid-term outcomes of colorectal ESD, and concluded that the colorectal ESD could be carried out effectively and safely with 89% en bloc resection rate, 76% R0 resection rate, and 94% endoscopic clearance rate.^[17]^ In previous reports from single-center studies, the en bloc resection rates ranged from 86.6% to 98.2%, and the curative resection rates ranged from 85.1% to 89.6%, which were consistent with our presented results.^[9–11,18,19]^

As for the factors related to the en bloc resection, several previous studies illustrated that, tumor size, submucosal fibrosis, invasive depth, and procedure time were associated with incomplete resection.^[9–11,18–20]^ In the present study, the univariate logistic regression analysis revealed that both tumor size and submucosal fibrosis were significantly associated with incomplete resection; while the multivariate logistic regression analysis identified that only submucosal fibrosis was the independent factor related to incomplete resection of colorectal ESD. As a matter of fact, ESD can be performed in en bloc manner irrespective of tumor size, however, in regard to tumor size as an independent factor affecting the en bloc resection of colorectal ESD, the results are still in controversy. The increasing improvement in devices and technique for ESD and different proficiency of endoscopists may explain the different results regarding to the risk associated with tumor size. In our present study, the mean colorectal neoplasm size was 26.5 mm, range from 15 to 60 mm, with a high en bloc resection rate of 93.6%. The explanation for the relatively higher en bloc resection rate for large neoplasms might be, firstly, that we included data from recent period, when the devices and technique for ESD were all advanced and mature in our department. Secondly, the three endoscopists in our study, were all have extensive training and professional experience in gastric ESD procedure, and were all skilled in various electric knife, hemostatic, and stitching instruments. Thirdly, the explanation might be the comprehensive preoperative evaluation of lesions and the selection of appropriate procedures. Therefore, we are confident that, as the constantly improvement of colorectal ESD equipment and technique, ESD has already enabled the en bloc resection for large colorectal superficial tumors.

Perforation is the main complication of colorectal ESD. Because of the much more thinner of the colonic wall, the risk of perforation during the procedure is higher than that of the stomach. The perforation rate of colorectal ESD was reported to range from 1.4% to 10.4%.^[3,6,7,9–11]^ In the present study, the perforation rate of colorectal ESD was 4.9%, and all cases were closed successfully with endoscopic clipping and following intravenous antibiotic conservative treatment. There was no additionally surgery for perforation, because perforations were tiny enough to be closed by clips and treated conservatively. Previous reports revealed that, larger size, location, invasive depth, fibrosis, and less experienced ESD colonoscopists were considered as the risk factors for perforation during colorectal ESD.^[6,7,9,11]^ Hong et al^[8]^ recently developed a score consisting of simple clinical factors, which based on tumor size, tumor location, endoscopist experience, and submucosal fibrosis, to estimate the risk of colorectal ESD-induced perforation. Similar variables have been analyzed in this present study, the univariate logistic regression analysis revealed that tumor size and submucosal fibrosis were significantly associated with perforation of colorectal ESD. While multivariate logistic regression analysis identified that submucosal fibrosis was the only independent factor related to perforation of colorectal ESD, which was consistent with the results reported previously.^[6–9,11]^ As for tumor size, which differs from the results of others, the different in colorectal ESD treatment periods may explain the difference, as well as the extensive trained and experienced colonoscopists.

In this present study, we have drew a conclusion that, in cases of lesions with submucosal fibrosis for colorectal ESD, the en bloc resection rate was low and the perforation rate was high, which were consistent with those in previous reports.^[10,11,13,21]^ As mentioned before, submucosal fibrosis is caused by the peristaltic motion or forceps biopsy, and severe fibrosis can complicate the separation of the submucosa from the muscular layer. In such cases, after injection the solution rapidly into submucosal layer, a blue transparent layer cannot be easily exfoliated in the ESD procedure. According to previous studies, we judged submucosal fibrosis on the basis of findings obtained at the time of submucosal dissection after the injection. In the present study, submucosal fibrosis was identified in 29.8% lesions during colorectal ESD. The en-bloc resection rate and the perforation rate for lesions with submucosal fibrosis in the present study were consistent with those in previous reports. From the histological point of view, Lee et al^[22]^ evaluated the histologic submucosal fibrosis of colorectal ESD samples through a pathologic review by using Masson's trichrome staining, which verified that fibrosis was the most powerful risk factor for en bloc resection and complications. Makino et al^[23]^ tried to predict the presence of fibrotic lesions preoperative in the colon by using endoscopic ultrasound sonography, which sensitivity and specificity were 77.8% and 57.1%. Hence, evaluation of submucosal fibrosis might be useful to predict the technical difficulties of colorectal ESD, and further studies should be focus on the preoperative factors predicting fibrosis in lesions treated by colorectal ESD. Therefore, lesions with submucosal fibrosis should be addressed cautiously by experienced endoscopists owing to the increased risk for incomplete resection and perforation.

In the present study, the en bloc resection rate was 93.6%, while the curative resection rate was 88.7%. The additional surgery rate was 2.8% and there was no recurrence occurred in all patients during the intensively follow periods. A multicenter prospective study stated that the local recurrence rates were 4.3% (65/1524), 6.8% (55/808), and 1.4% (10/716) for the entire cohort, for piecemeal EMR, and for ESD, which demonstrated that piecemeal resection was the most important risk factor for local recurrence.^[24]^ Our outcomes indicated that ESD significantly reduced the local recurrence rate of large colorectal neoplasms, but follow up should be pay attention to piecemeal resected lesions intensively.

Indeed, our study still has some limitations. Firstly, it was a retrospective, single-center study. Secondly, our study included a relatively small sample size; thirdly, the extent of the tissue fibrosis in our study was assessed by endoscopists based on endoscopic findings, not by pathological findings. Prospective studies including larger number of patients are needed to confirm our results.

In conclusion, we confirm that ESD is a very safe and effective technique for en bloc resection of large superficial colorectal neoplasms. Colorectal ESD is becoming increasingly standardized technique worldwide. Submucosal fibrosis is an independent risk factor for both incomplete resection and perforation of colorectal ESD, and the evaluation of submucosal fibrosis might be useful to predict the technical difficulties of colorectal ESD. Further improvement will be required for safe and complete resection of colorectal neoplasms with submucosal fibrosis.

## Acknowledgments

The authors wish to thank the endoscopy staff for their assistance.
